# Identification of Polyvalent Vaccine Candidates From Extracellular Secretory Proteins in *Vibrio alginolyticus*

**DOI:** 10.3389/fimmu.2021.736360

**Published:** 2021-10-04

**Authors:** Yu-ming Peng, Jian-jun Tao, Su-fang Kuang, Ming Jiang, Xuan-xian Peng, Hui Li

**Affiliations:** ^1^ State Key Laboratory of Bio-Control, School of Life Sciences, Southern Marine Science and Engineering Guangdong Laboratory (Zhuhai), Sun Yat-sen University, University City, Guangzhou, China; ^2^ Laboratory for Marine Fisheries Science and Food Production Processes, Qingdao National Laboratory for Marine Science and Technology, Qingdao, China

**Keywords:** vaccine, polyvalent vaccine, DNA vaccine, extracellular secretory proteome, bacteria

## Abstract

Bacterial infections cause huge losses in aquaculture and a wide range of health issues in humans. A vaccine is the most economical, efficient, and environment-friendly agent for protecting hosts against bacterial infections. This study aimed to identify broad, cross-protective antigens from the extracellular secretory proteome of the marine bacterium *Vibrio alginolyticus*. Of the 69 predicted extracellular secretory proteins in its genome, 16 were randomly selected for gene cloning to construct DNA vaccines, which were used to immunize zebrafish *(Danio rerio).* The innate immune response genes were also investigated. Among the 16 DNA vaccines, 3 (AT730_21605, AT730_22220, and AT730_22910) were protective against *V. alginolyticus* infection with 47–66.7% increased survival compared to the control, while other vaccines had lower or no protective effects. Furthermore, AT730_22220, AT730_22910, and AT730_21605 also exhibited cross-immune protective effects against *Pseudomonas fluorescens* and/or *Aeromonas hydrophila* infection. Mechanisms for cross-protective ability was explored based on conserved epitopes, innate immune responses, and antibody neutralizing ability. These results indicate that AT730_21605, AT730_22220, and AT730_22910 are potential polyvalent vaccine candidates against bacterial infections. Additionally, our results suggest that the extracellular secretory proteome is an antigen pool that can be used for the identification of cross-protective immunogens.

## Introduction

Aquaculture presents an opportunity to increase food production in the face of a growing demand for high protein diets (1). However, the increasing development of finfish aquaculture has affected the environment and human health. These effects are related to the widespread and unrestricted use of antibiotics as additives in the fish food industry, especially in developing countries (2). The unchecked use of antibiotics leads to their accumulation in the aquatic environment and aquatic products, as well as the emergence of antibiotic-resistant bacteria (3, 4). Metabolite-enabled killing of antibiotic-resistant bacteria by antibiotics and hosts has recently been developed as an efficient approach against pathogens with a reduced dose of antibiotics and without antibiotics, respectively (5–15). Alternatively, the use of vaccines has emerged as a superior strategy to cope with bacterial infection without using antibiotics (16–18).

The rate of development of effective vaccines is unable to meet their growing demand in aquaculture. Two major challenges impeding this process are the combination of different species of pathogens and the inconvenience of multiple injections (19–21). Aquatic pathogens belong to a variety of species from different genera and families (22). Vaccines may be monovalent, multivalent, or polyvalent. Both monovalent and polyvalent vaccines come from protein antigens, and multivalent vaccines are mixed with at least two monovalent vaccines. Monovalent vaccines specifically target one of the pathogens, while multivalent and polyvalent vaccines protect hosts against infections caused by at least two pathogens. To protect infections caused by more than two pathogens, multiple injections of monovalent vaccines are needed (23, 24). Multiple injections are inconvenient and uneconomical, especially in China, where cultured fish are small in size and thus multiple injections are not feasible. A single injection of a multivalent vaccine contains two or more monovalent vaccines, but the resulting immune protection is usually reduced because different vaccines together may interfere with each other (24, 25). However, polyvalent vaccines against more than one species of pathogens are a still more promising strategy to overcome the current limitations and can be a more efficient and economical method (19–21). Thus, the development of effective polyvalent vaccines is in high demand.

Recently, we identified several polyvalent vaccine candidates from the outer membrane proteins of pathogenic bacteria. The vaccines VP1061, VP2850, VP2309, VP0887, VPA0548, and VP1019 from *Vibrio. parahaemolyticus* conferred protection against infection with *V. parahaemolyticus* and two other aquaculture pathogens, *P. fluorescens* and *A. hydrophila* (20, 21). Furthermore, polyvalent OmpA vaccines against *Edwardsiella tarda* and *Vibrio* were constructed by DNA shuffling of five *ompA* genes from four species of bacteria, *E. tarda, V. parahaemolyticus, V. alginolyticus*, and *Escherichia coli.* The polyvalent OmpA vaccines protected zebrafish against infections caused by *E. tarda* and *V. alginolyticus*, suggesting that molecular breeding *via* DNA shuffling directs the evolution of polyvalent vaccines with desired traits (26–29). We also identified immunogens from bacterial extracellular secretory proteins and found that the secretory proteins represent a novel pool of targets to search for antigens inducing immune protective response (30).

In this study, 16 extracellular secretory protein genes from *V. alginolyticus* genome were randomly selected for cloning and construction of DNA vaccines, and their protective efficacy was evaluated in a zebrafish model using an active immunization approach. Three of the 16 genes, AT730_21605, AT730_22220, and AT730_22910, were identified as highly cross-protective antigens.

## Materials and Methods

### Bacterial Strains and Culture Conditions

Strains of *V. alginolyticus, P. fluorescens*, and *A. hydrophila* were cultured in our laboratory. These bacteria were grown at 28°C in Luria-Bertani (LB) medium. To propagate the bacterial culture, a single colony from each strain was picked and cultured in fresh LB medium at 28°C overnight. The cultures were then diluted at 1:100 with fresh LB medium and grown to an OD_600_ of 1.0 at 200 rpm at 28°C.

### Experimental Fish

Zebrafish (*Danio rerio*, 0.3 g average weight) were purchased from Shaoping Corp., Guangzhou Huangsha, China. Before using in experiments, the fish were acclimated in 25 L open-circuit water tanks with aeration for two weeks and tested free of *Vibrio* species, *A. hydrophila* and *P. fluorescens* through zebrafish homogenates cultured in thiosulphate‐citrate‐bile salts‐sucrose agar medium (TCBS) for *Vibrio* and glutamate starch phenol red agar medium (GSP) for *Pseudomonas* and *Aeromonas*, respectively. The fish were fed a balanced commercial feed (Hikari Tropical Fancy Guppy, Kyorin, Hyogo, Japan) at a ratio of 3% of body weight per day. The study was approved by the Institutional Animal Care and Use Committee of Sun Yat-sen University (approval No. SYSU-IACUC-2020-B126716).

### Prediction of *V. alginolyticus* Extracellular Proteins

The online software PSORTb v 3.0.2 (https://www.psort.org/psortb/) was used to analyze subcellular locations. The amino acid sequences of all 4698 proteins of *V. alginolyticus* (ATCC 33787) were downloaded from Swiss-Prot and searched against PSORTb. Protein sequences were analyzed, and their subcellular locations were scored. The score was used to predict the potential location of a protein. Proteins predicted to be in extracellular location or in multiple locations with an extracellular score over 1.0, were considered to be extracellular proteins.

### Gene Cloning and Prokaryotic Expression Plasmids

For gene cloning, 16 genes were randomly selected and their respective primer pairs were designed (**Table 1**) according to *V. alginolyticus* ATCC 33787 sequences released by GenBank (NC_022349.1, NC_022359.1). *V. alginolyticus* genomic DNA was used as a template for standard PCR. The PCR products were purified and digested using restriction endonucleases. The fragments were cloned into the expression vector pET-32a with T4 DNA ligase. The recombinant plasmids were transformed into *E. coli* DH5α and the recombinants were screened on LB agar solid medium with 100 μg/mL ampicillin. The plasmids were extracted, transformed into *E. coli* BL21 (DH3) and screened as described above. The DH3 recombinants were cultured in LB medium at 37°C, 200 rpm. Isopropyl-b-D-thiogalactoside (IPTG) was added to the final concentration of 0.1 mM when bacteria grew to OD_600_ = 0.6. The cultures were harvested after 5 h, and recombinant proteins were analyzed by SDS-PAGE.

### Construction and Screening of DNA Vaccines

DNA vaccines were constructed and screened according to a previously described protocol (19, 20). The eukaryotic expression vector pcDNA3.1(+) (Novagen) was digested with restriction enzymes *BamH*I and *Xho*I. The 16 genes treated with the same restriction enzymes were ligated to pcDNA3.1(+). For obtaining eukaryotic expression plasmids, the correct recombinant plasmids were selected by routine restriction endonuclease identification. The DNA plasmids were purified by the QIAGEN plasmid purification kits and then the endotoxin-free plasmid DNA was obtained by Endo-Free Plasmid DNA Midi Kit (Omega) as DNA vaccines.

### Western Blot for Expression of DNA Vaccines in Fish

The zebrafish were randomly divided into four groups, with five fish in each group. They were intramuscularly injected with 1.5 μg DNA vaccine AT730_21605, AT730_22220, or AT730_22910, and pCDNA3.1(+) vector was used in the control group. Two weeks post-immunization, three fish were randomly selected from each group. After trimming the head and visible fat surrounding the abdomen, these animals were separately homogenized in cold PBS and kept at 4°C overnight. Supernatants were collected by centrifugation at 12,000 rpm. Proteins in the supernatants were separated by SDS-PAGE and transferred to polyvinylidene fluoride (PVDF) membranes using a constant voltage of 80 V for 1 h at 4°C. The membranes were stained with Ponceau S to evaluate the transfer efficiency and then blocked for 1 h in 5% non-fat milk in Tris-buffered saline and Tween 20 (TBST) buffer at 4°C. The blocked membranes were incubated with mouse antisera against AT730_21605, AT730_22220, or AT730_22910 (prepared in our lab) for 1 h on a gentle shaker at 37°C. After washing three times for 10 min with TBST buffer, the membranes were incubated with rabbit anti-mouse horseradish peroxidase (HRP)-conjugated secondary antibodies (Xiamen Bosheng Biotech. Corp. China) for 1 h under identical conditions. The membranes were washed and developed with Super-Lumia ECL plus HRP Substrate Kit (Abbkine, ABB-K22030) in a G:BOX (Syngene, England) system until maximum color appeared.

### Immunization and Challenge

Zebrafish were randomly divided into 17 groups, with 20 fish in each group, for the selection of highly protective vaccine candidates against *V. alginolyticus* infection. Each fish was intramuscularly injected with 1.5 μg DNA vaccine (31). The pCDNA3.1(+) vector was injected into the control group. For the cross-protection study, zebrafish were randomly divided into 8 groups, with 20 fish in each group. They were separately immunized with 1.5 μg AT730_21605, AT730_22220, AT730_22910 in experimental groups and pCDNA3.1(+) vector in control groups. These fish were cultured at 28°C and fed twice daily with commercial feed for 4 weeks until being exposed to *V. alginolyticus* with 1.6 × 10^6^ CFU/fish, *A. hydrophila* with 4 × 10^5^ CFU/fish, *and P. fluorescens* with 1.6 × 10^6^ CFU/fish. These doses were obtained by LD50 evaluation. The fish were observed for 15 days to measure their relative percent survival (RPS). The formula used for calculating the RPS index was as follows: 1 - (% vaccinated mortality/% non-vaccinated mortality) × 100. Differences between groups were tested for significance using the statistical analysis software SPSS for small numbers at two significance levels (0.05 and 0.01).

### Phylogenetic Analysis

Phylogenetic analysis was performed as previously described (29). AT730_21605, AT730_22220, and AT730_22910 were separately compared with the NCBI database (https://www.ncbi.nlm.nih.gov/) using the blastp algorithm. A multiple sequence alignment was created on amino acid sequences of known proteins from other bacterial species using Clustal W. Phylogenetic trees were constructed based on these sequences using MEGA7 through the neighbor-joining (NJ) method. Bootstrap tests with 1,000 replicates were performed to examine the validity of the branching topologies. B epitope prediction was analyzed using ANTIGENIC from EMBOSS (http://www.bioinformatics.nl/cgi-bin/emboss/antigenic). Alignment results produced by Vector NTI 11 and the secondary structure were predicted by SOPMA (https://npsa-prabi.ibcp.fr/cgi-bin/npsa_automat.pl?page=npsa_sopma.html).

### qRT-PCR for Measuring Expression of Innate Immune Genes

The zebrafish were randomly divided into 4 groups, with 20 fish in each group. They were intramuscularly injected with 1.5 μg DNA vaccines, and pCDNA3.1(+) vector was used in the control group. Whole head kidney, liver, spleen and heart per fish were extracted and pooled, which were collected on the 5^th^ day and 10^th^ post-immunization (32). Four biological samples were performed in each group. Total RNA was isolated from these samples using TRIzol reagent (Invitrogen, USA). The RNA was quantified spectrophotometrically. qRT-PCR was carried out on 1 μg of total RNA using a SYBR Green Premix *Pro Taq* HS qPCR Kit with a gDNA eraser (Accurate Biotechnology, China) according to the manufacturer’s instructions. It was performed in 384-well plates with a total volume of 10 μL containing 5 μL 2 × SYBR Premix Ex Taq™, 2.2 μL PCR-grade water, 2 μL cDNA template, and 0.4 μL each of forward and reverse primers (10 μM). All primers used for qPCR are shown in **Supplementary Table 1**. *EF-1α* was used as the reference gene (33). All samples were assayed in triplicate and run on a CFX384-Touch qPCR system (Bio-Rad, USA) according to the manufacturer’s instructions. The cycling parameters were as follows: 95°C for 30 s to activate the polymerase, 40 cycles of 95°C for 5 s, 58°C for 30 s, and fluorescence measurements were performed at 72°C for 1 s during each cycle. Cycling was terminated at 95°C with a calefactive velocity of 5°C/s to obtain a melting curve. To analyze the relative expression levels of target genes, we converted the data to percentages relative to the value of the pCDNA3.1(+) vector control.

### Neutralizing ELISA for Measuring Neutralizing Ability

The zebrafish were randomly divided into 4 groups with 20 fish in each group. They were intramuscularly injected with eukaryotic plasmids AT730_21605, AT730_22220, AT730_22910 and pCDNA3.1(+) for the collection of antigen-immunized plasma and pCDNA3.1(+)-immunized plasma, respectively. The heart of zebrafish was surgically obtained on the 20^th^ day post the injection and blood was collected and pooled in 3% heparin solution (plasma). Bacterial protein samples were separated by 10% SDS-PAGE and transferred to nitrocellulose membranes (NC) under the condition of constant voltage of 80 V for 1 h. The membranes were blocked with 5% non-fat milk powder at 37°C for 2 h and then cut for three groups, control group with TBST buffer (group 1), control group with pCDNA3.1(+)-immunized plasma (group 2), and neutralizing group with antigen-immunized plasma (group 3). Subsequently, group 1, group 2, and group 3 were incubated with 5% non-fat milk powder, 1:10 of pCDNA3.1(+)-immunized and antigen-immunized plasma diluted by 5% non-fat milk powder, respectively, at 37°C for 2 h. After washed three times for 10 min with TBST buffer, the membranes were incubated with 1:200 of mouse antibodies against AT730_21605, AT730_22220, or AT730_22910 at 37°C for 2h. Then, the membranes were incubated with 1:3,000 of rabbit anti-mouse antibodies at 37°C for 1 h followed by washing three times for 10 min with TBST buffer. The protein bands on the nitrocellulose membranes were detected by enhanced chemiluminescence substrate on imaging analysis system, Tanon-5200. Neutralizing rate was calculated by 1 – (group 3/group 1).

## Results

### Identification of Extracellular Secretory Proteins

Using PSORTB 3.0.2, 69 proteins were predicted to be extracellular secretory proteins because their location scores were high for extracellular proteins but low for cytoplasmic membrane proteins, periplasmic proteins, cytoplasmic proteins, and outer membrane proteins (**Supplementary Table 2**).

### Cloning of Genes and Expression of Prokaryotic Recombinant Proteins

**Figure 1:**
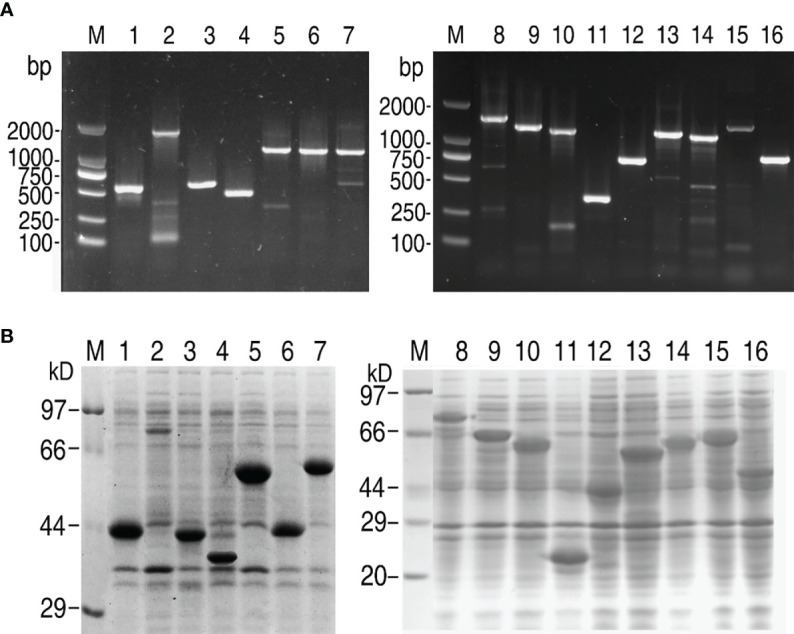
Gene cloning and SDS-PAGE analysis of *V. alginolyticus* extracellular proteins. **(A)** PCR for gene cloning. **(B)** SDS-PAGE analysis for prokaryotic expression of targeted proteins. M: DNA/protein markers; Lanes 1-16: AT730_04380; AT730_06145; AT730_07035; AT730_08185; AT730_14920; AT730_14930; AT730_14935; AT730_19120; AT730_19300; AT730_20810; AT730_20950; AT730_21050; AT730_21605; AT730_22220; AT730_22910; AT730_24225.

Sixteen secretory protein genes (**Supplementary Table 3**) were randomly selected and successfully amplified. The resulting DNA fragments were identified and purified by 1% agarose electrophoresis (**Figure 1A**). The gene fragments were cloned into the expression vector pET-32a(+) plasmid. To confirm amplification, these DNA fragments were digested with restriction endonucleases (**Supplementary Figure 1**). The recombinant plasmids were transformed into *E. coli* BL21 (DH3) and screened by growth on LB plates containing ampicillin. Colonies were picked and cultured in LB medium, and then IPTG was added to induce the expression of recombinant proteins. The recombinant proteins containing a 20 kDa fusion tag were checked by SDS-PAGE to identify whether these cloned genes could be expressed. Only one band at the corresponding molecular mass was observed for all genes (**Figure 1B**). These data showed that these genes were successfully cloned and expressed for further investigation.

### Construction of DNA Vaccines

The 16 genes were used to construct a eukaryotic expressing library using the eukaryotic expression vector pcDNA3.1(+) to construct DNA vaccines. Recombinants were identified by double-enzyme digestion. The results from digestion confirmed that 16 DNA vaccines were successfully constructed (**Supplementary Figure 2**).

### Protective Ability of DNA Vaccines in a Zebrafish Model

**Figure 2:**
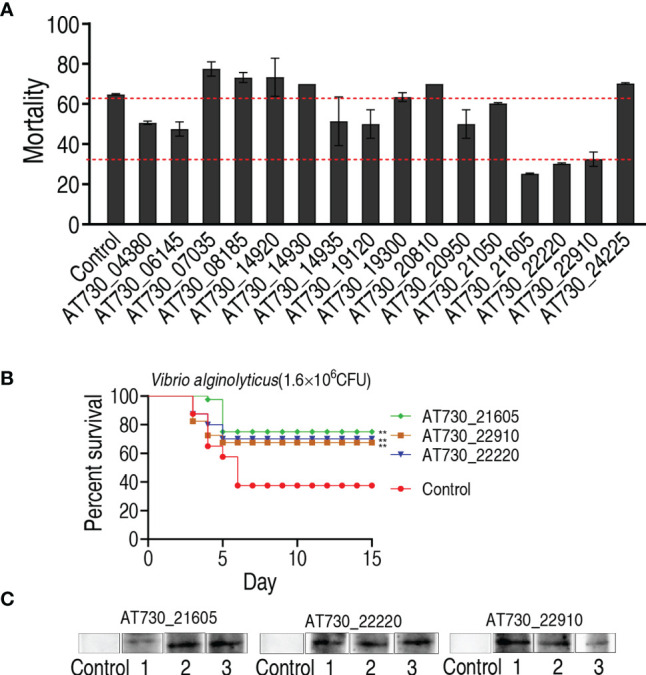
Active immune protection against *V. alginolyticus* in zebrafish. **(A)** Active immunization with DNA vaccines and challenged by *V. alginolyticus* with 1.6 × 10^6^ CFU/fish. pcDNA3.1(+) vector is used as control. **(B)** Percent survival of zebrafish separately immunized with AT730_21605, AT730_22220 and AT730_22910 and challenged by *V. alginolyticus* with 1.6 × 10^6^ CFU/fish. The challenge experiments were repeated twice. **(C)** Western blot for expression of DNA vaccines in zebrafish at 2 weeks post DNA vaccination. Vector pcDNA3.1(+) is used as control.

To investigate the immune protective ability of the 16 DNA vaccines, zebrafish were randomly divided into 17 groups, with 20 fish in each group. They were intramuscularly injected with one of the 16 DNA vaccines in the 16 test groups and with vector pcDNA3.1(+) in the negative control group. The fish were intramuscularly exposed to *V. alginolyticus* (1.6 × 10^6^ CFU/fish) for 4 weeks post-immunization. Protection was assessed by determining the accumulated death of zebrafish at 15 days post-immunization. Among the 16 DNA vaccines, nine, AT730_04380, AT730_06145, AT730_14935, AT730_19120, AT730_20950, AT730_21050, AT730_21605, AT730_22220, and AT730_22910, showed significant immune protection against bacterial infection compared with the negative control (**Figure 2A**). Importantly, three vaccines, AT730_21605, AT730_22220, and AT730_22910, showed 66.7%, 50%, and 47% RPS, respectively, and thus were selected as highly protective vaccine candidates against *V. alginolyticus* infection and used for further identification of broad cross-immune protection candidates (**Figure 2B**). In addition, three fish were randomly collected on day 5 in the groups immunized with AT730_21605, AT730_22220, or AT730_22910. Western blotting using specific antibodies against AT730_21605, AT730_22220, and AT730_22910 revealed a specific band in each fish, but not in the control **(** **Figure 2C** **)**, indicating that these DNA vaccines were expressed in fish.

### Cross-Protective Ability of DNA Vaccines in a Zebrafish Model

**Figure 3:**
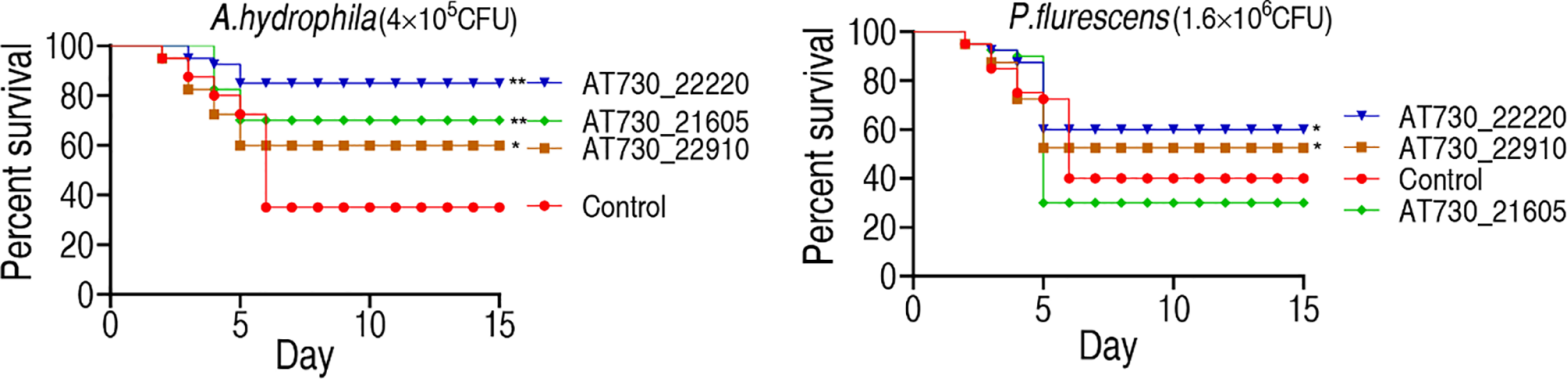
Active immune protection against *A. hydrophila* and *P. fluorescens* in zebrafish. Zebrafish were separately immunized with DNA vaccines and challenged by *A. hydrophila* with 4 × 10^5^ CFU/fish and by *P. fluorescens* with 1.6 × 10^6^ CFU/fish. The formula of calculating the RPS index is as below: 1 - (% vaccinated mortality/%non-vaccinated mortality) × 100.

Zebrafish were randomly divided into groups and immunized with AT730_21605, AT730_22220, or AT730_22910. They were exposed to *A. hydrophila* (4 × 10^5^ CFU/fish) or *P. fluorescens* (1.6 × 10^6^ CFU/fish) 4 weeks post-immunization. The fish were observed for 15 days to monitor their RPS. The protective immunogens showed differential cross-protective effects. Specifically, AT730_21605, AT730_22220, and AT730_22910 were 52.4%, 76.2%, and 36.5%, respectively, against *A. hydrophila* infection. However, AT730_22220 and AT730_22910 exhibited 33.33% and 28.6% RPS against infection with *P. fluorescens*, but AT730_21605 displayed no difference compared with the control (**Figure 3**). These results indicate that the three DNA vaccines showed cross-immune protection.

### Mechanisms for Cross-Protective Ability Based Conserved Epitopes

**Figure 4:**
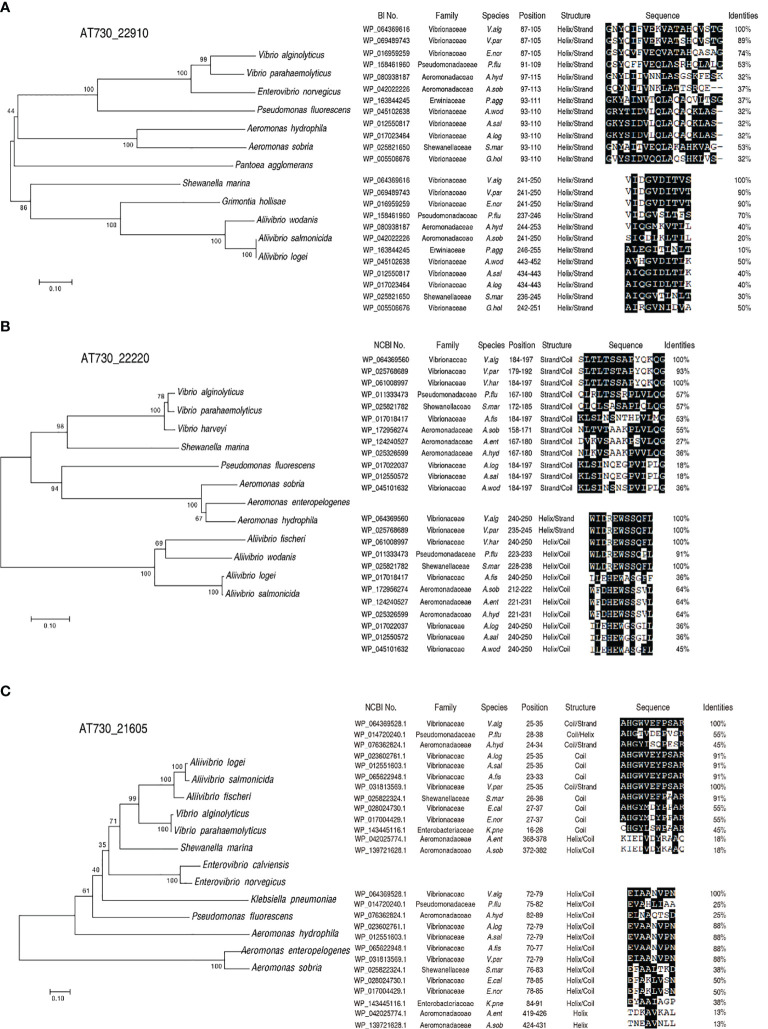
Construction of phylogenetic trees and blast analysis of epitopes. A phylogenetic tree with bootstrap values based on the full-length protein sequences of AT730_22910 **(A)**, AT730_22220 **(B)** and AT730_21605 **(C)**. The tree was constructed using a neighbor-joining method (left). B epitope prediction was analyzed using ANTIGENIC from EMBOSS (http://www.bioinformatics.nl/cgi-bin/emboss/antigenic) (right).

Alignment and phylogenetic tree construction were used to understand cross-protective mechanisms based on conserved epitopes. The peptide sequences of AT730_22220, AT730_22910, and AT730_21605 were obtained from GenBank. BLAST homology searches yielded matches of these peptide sequences with those of other bacteria. Specifically, phylogenetic trees showed that the three genes were conserved among *V. alginolyticus*, *A. hydrophila*, and *P. fluorescens* (**Figure 4**). Further analysis indicated that two conserved epitopes were separately detected among the bacteria in the three genes. Compared to *V. alginolyticus*, identity of the two epitopes of AT730_22910 is 53% and 70% in *P. fluorescens* and 37% and 40% in *A. hydrophila*, identity of the two epitopes of AT730_22220 is 57% and 91% in *P. fluorescens* and 38% and 64% in *A. hydrophila*, identity of the two epitopes of AT730_22220 is 55% and 25% in *P. fluorescens* and 45% and 25% in *A. hydrophila*. Thus, the identity of at least one epitope is equal or more than 40% in the three genes among the three pathogens. Most of these were helical epitopes. These results suggest that these homologous epitopes provide a basis for the cross-protection of the two vaccines.

**Figure 5:**
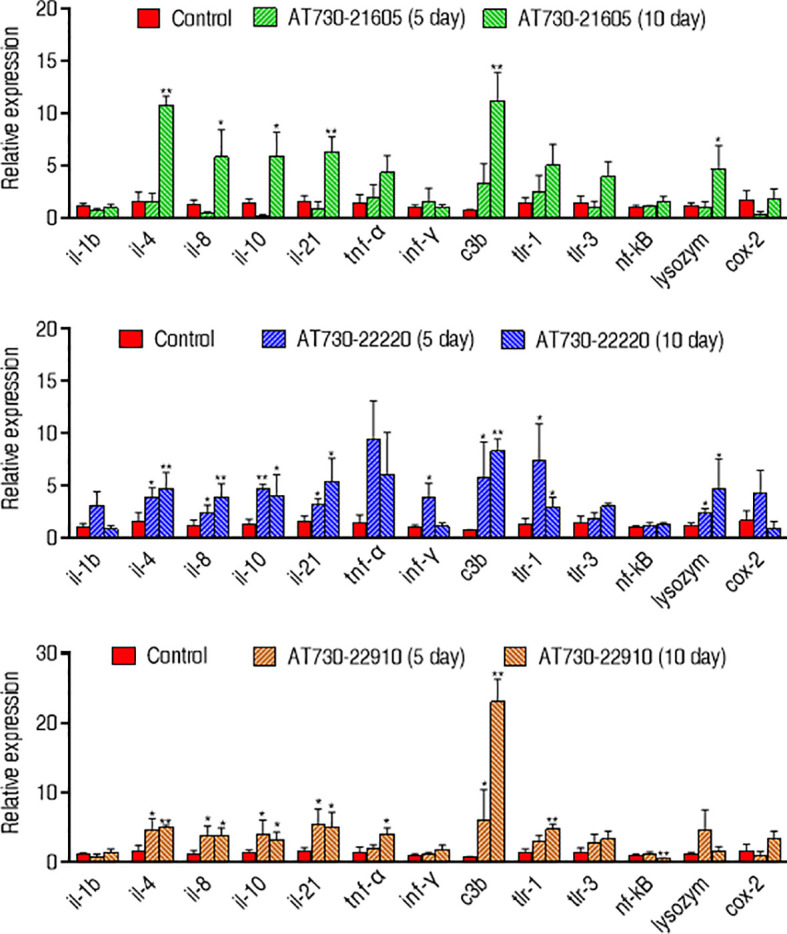
qRT-PCR for expression of innate immune genes. Whole head kidney, liver, spleen, and heart each fish were extracted and pooled on the 5th day and 10th day post-immunization. Four biological samples were performed in each group. mRNA was extracted for qRT-PCR. Results are displayed as mean ± SD. Fold increases were calculated relative to the expression level of the same genes in fish injected with control pCDNA3.1(+). Significant differences are identified (*P < 0.05; **P < 0.01) as determined by two-tailed Student’s t test.

Moreover, qRT-PCR was used to compare innate immune responses in the three groups immunized with AT730_21605, AT730_22220, or AT730_22910. Among the 12 genes detected, *il-1b, c3b, lysozyme*, and *cox-2* were elevated between the three experimental groups and the control. However, the other genes showed differences among the three experimental groups. Specifically, compared with the control, the AT730_21605-immunized group showed elevated *il-4*, *il-8, il-10, il-21*, C3b, and lysozyme, normal *il-1b*, *tnf-α*, *ifn-γ, tlr-1, tlr-3, nf-kB and cox-2;* AT730_22220-immunized group showed elevated *il-4*, *il-8, il-10, il-21*, *ifn-γ*, C3b, *tlr-1*, and lysozyme, normal *il-1b*, *tnf-α*, *tlr-3, nf-kB and cox-2;* AT730_22910-immunized groups showed elevated *il-4*, *il-8, il-10, il-21*, *tnf-α*, C3b, and *tlr-1*, normal *il-1b*, *ifn-γ, tlr-3*, lysozyme, *and cox-2*, low *nf-kB*. Almost all gene expression increased with the detected days (**Figure 5**).

**Figure 6:**
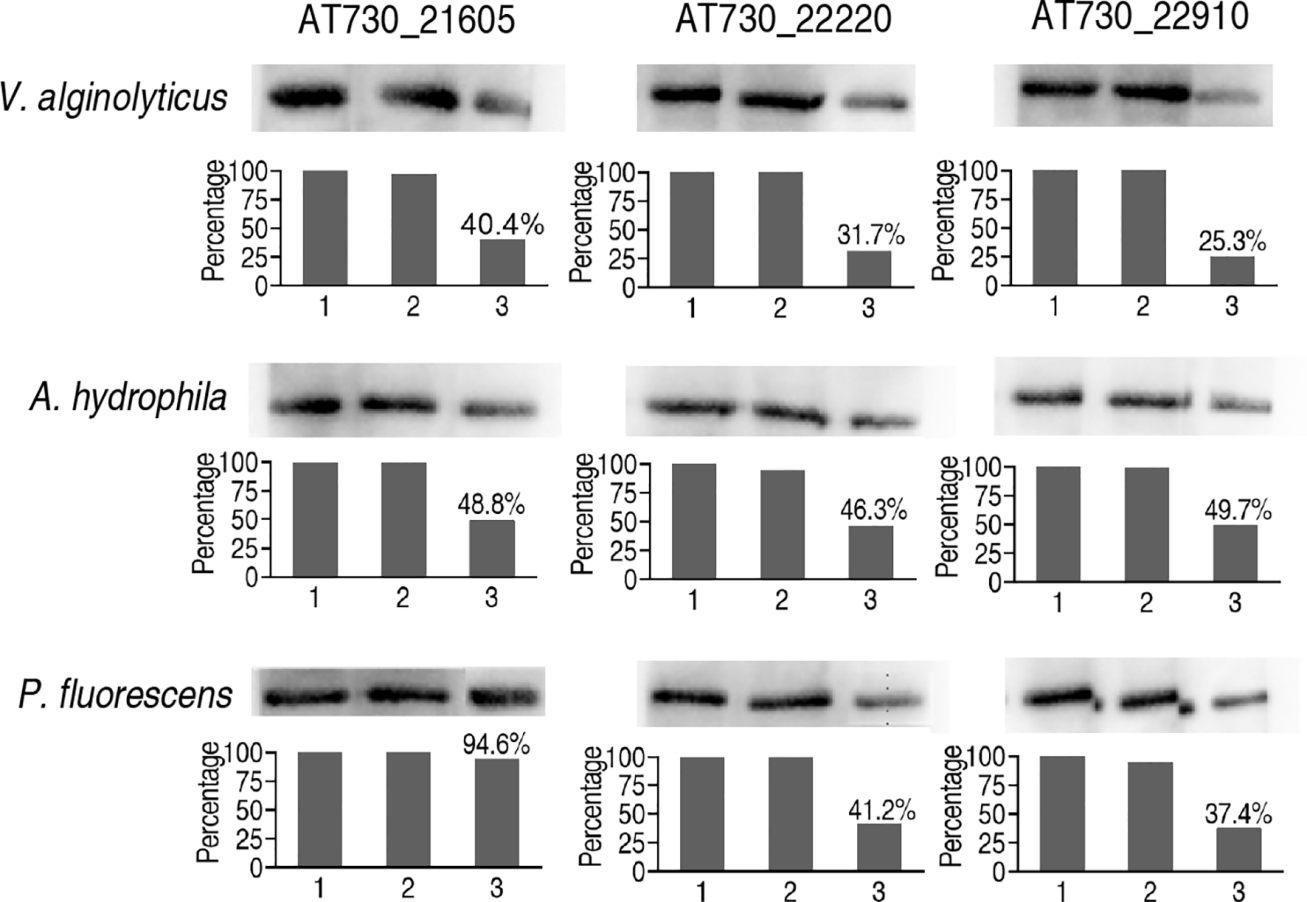
Neutralizing ELISA for neutralizing ability of the antibodies to the three DNA vaccines. The hearts of 20 zebrafish were surgically obtained on the 20^th^ day post the injection of AT730_21605, AT730_22220, AT730_22910, pCDNA3.1(+), and saline solution. Blood was collected and pooled in 3% heparin solution (plasma). Bacterial proteins were separated by SDS-PAGE and transferred to NC membranes. The NC membranes were cut and incubated with the plasma from the animals injected with solution (1), pCDNA3.1(+) (2), and AT730_21605, AT730_22220 or AT730_22910 (3). The membranes were incubated with mouse antiserum against AT730_21605, AT730_22220 or AT730_22910, respectively and then reacted with rabbit antiserum against mouse IgG. Neutralizing rate is calculated by 1 – [(3)/(1)].

Finally, neutralizing ELISA was adopted to detect neutralizing antibodies to AT730_22220, AT730_22910, and AT730_21605 in zebrafish injected with each of DNA vaccines of the three antigens. All neutralizing ability was higher than 50% in the immune protection and cross-protection except for the use of *P. fluorescens* and AT730_22220-immunized plasma as the neutralizing antigen and antibody, respectively (**Figure 6**).

## Discussion

Protective immunogens have been derived from secretory proteins and extracellular secretome (30), but whether the extracellular secretome contains cross-protective immunogens remains uncertain. The present study not only screened protective immunogens from the *V. alginolyticus* extracellular secretome but also identified cross-protective immunogens from these candidates. Therefore, these results indicate that the *V. alginolyticus* extracellular secretome is an ideal source for the identification of cross-protective immunogens against bacteria in aquaculture.

In contrast to monovalent vaccines, which show immune protective ability against one type of bacteria (23, 24), polyvalent vaccines display cross-protective ability against more than one type of bacteria (19–21). This is particularly beneficial in aquaculture because the cultured fish are small in size and multiple injections of monovalent vaccines to prevent bacterial infection are not well received in developing countries such as China. Thus, the administration of a polyvalent vaccine is an efficient approach to control bacterial infections (19, 20). The present study shows that zebrafish immunized with AT730_22220 or AT730_22910 exhibited the ability to survive from *V. alginolyticus*, *A. hydrophila*, and *P. fluorescens* infections, while the animals immunized by AT730_21605 displayed the capability to survive infection with *V. alginolyticus* and *A. hydrophila*. The cross-protective immunity of the other 13 vaccines did not investigated due to their lower or no protection against *V. alginolyticus.* AT730_21605 is a lytic polysaccharide monooxygenase (LPMO), which is a copper-dependent enzyme involved in the degradation of recalcitrant polysaccharides such as cellulose or chitin. LPMOs act synergistically with glycoside hydrolases such as cellulases and chitinases by oxidatively cleaving several glycosidic bonds at the surface of their crystalline substrate(s). In addition to their role in biomass degradation, some bacterial LPMOs act as virulence factors in several human and insect pathogens (34). AT730_22220 is carotenoid 1,2-hydratase (CrtC), which catalyzes the selective addition of water to an isolated carbon-carbon double bond (35). AT730_22910 is a flagellar filament capping protein (FliD), which is found at the distal end of the flagellar filament as a flagellar cap (36, 37). FliD is a structural protein that protects the flagellum tip. FliD is also a functional protein that repetitively inserts flagellin proteins to extend the flagellar filaments (38). The present study shows that these three proteins are cross-protective immunogens. Reports have shown that hpFliD has been developed as a promising vaccine target that protects humans from *Helicobacter pylori* infection (39, 40). To our knowledge, information regarding the cross-immune protection of these proteins is lacking. Therefore, this is the first study to reveal the cross-immune protective effects of these proteins.

To understand the mechanisms by which the three DNA vaccines show cross-protective ability against the three pathogens, bioinformatics analysis was used to characterize conserved epitopes of the three proteins among the bacteria. This analysis shows that the three proteins of *V. alginolyticus* have a close relationship with those of other bacteria, including *A. hydrophila* and *P. fluorescens*, where two conserved epitopes were detected among these bacteria. Thus, these conserved epitopes may contribute to cross-protective immunity, supporting the conclusion that polyvalent immunogens are usually highly conserved (19, 20). Furthermore, the present study explored possible cross-protective mechanisms by detecting the innate immune response to the three cross-protective immunogens. Elevation of *il-4*, *il-8, il-10, il-21*, and C3b overlapped among the zebrafish immunized by the three DNA vaccines. Among the five elevated genes, *c3b* are humoral innate immune mediators, *il-8* plays a key role as pro-inflammatory molecules in fish (41, 42), *il-4*, *il-10*, and *il-21* supporting B-cell and T-cell development and activation (43–45). Therefore, the overlapped elevation may contribute to the cross-protective ability of *V. alginolyticus* and *A. hydrophila*. At last, specific antibodies to AT730_21605, AT730_22220, and AT730_22910 were investigated by neutralizing ELISA. All neutralizing ability was higher than 50% except for the neutralization to *P. fluorescens* antigen by plasma immunized with AT730_21605, which was consistent to the earlier result that the cross-protection was not detected in zebrafish immunized with AT730_21605 and then challenged by *P. fluorescens* (**Figure 3**). Thus, specific antibodies play a role in the immune protection and cross-protection.

In summary, the present study investigated 16 DNA vaccines randomly selected from the secretome of *V. alginolyticus*. Nine of the vaccines showed significant immune protection against bacterial infections. Among them, AT730_21605, AT730_22220 and AT730_22910 showed 66.7%, 50% and 47% RPS, respectively. Furthermore, cross-immune protection against *A. hydrophila* and *P. fluorescens* infection was investigated in the three vaccines. AT730_22220 and AT730_22910 provided protection against infection with the two pathogens, whereas AT730_21605 conferred protection only against *A. hydrophila* but not against *P. fluorescens*. Finally, conserved epitopes, overlapped elevation of *il-4*, *il-8, il-10, il-21*, and C3b and neutralizing and cross-neutralizing experiments were detected among these bacteria. These results highlight a novel method to identify polyvalent vaccine candidates from the secretome.

## Data Availability Statement

The original contributions presented in the study are included in the article/**Supplementary Material**. Further inquiries can be directed to the corresponding author.

## Ethics Statement

The animal study was reviewed and approved by the Institutional Animal Care and Use Committee of Sun Yat-sen University (Approval NO. SYSU-IACUC-2020-B126716).

## Author Contributions

HL and X-XP conceptualized and designed the project. Y-MP, J-JT, MJ, and S-FK performed the experiments. Y-MP and HL interpreted the data. HL and X-XP wrote the manuscript. All authors contributed to the article and approved the submitted version.

## Funding

This work was sponsored by grants from Guangzhou Science and Technology Project (201904020042), Innovation Group Project of Southern Marine Science and Engineering Guangdong Laboratory (Zhuhai) (No. 311021006), and International Exchanges Scheme (NSFC-RS, 31911530183).

## Conflict of Interest

The authors declare that the research was conducted in the absence of any commercial or financial relationships that could be construed as a potential conflict of interest.

## Publisher’s Note

All claims expressed in this article are solely those of the authors and do not necessarily represent those of their affiliated organizations, or those of the publisher, the editors and the reviewers. Any product that may be evaluated in this article, or claim that may be made by its manufacturer, is not guaranteed or endorsed by the publisher.
