# Supplementary material for: Identification of Polyvalent Vaccine Candidates From Extracellular Secretory Proteins in Vibrio alginolyticus
Source: Front Immunol. 2021 Oct 4;12:736360. doi: 10.3389/fimmu.2021.736360 (PMC8521057; doi:10.3389/fimmu.2021.736360)
Supplement: Supplementary file 1 [file Presentation_1.pdf]

Supplementary Material

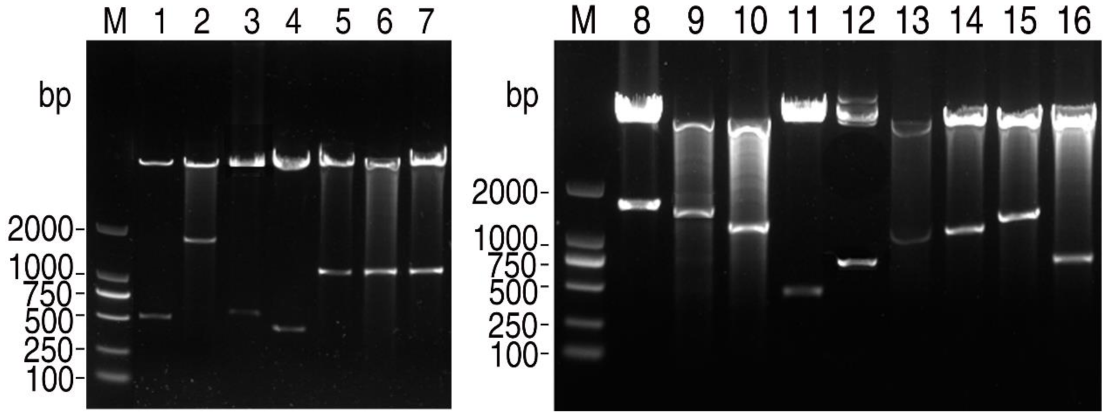

Supplementary Figure 1. Identification of prokaryotic expression plasmid by restriction enzyme analysis.

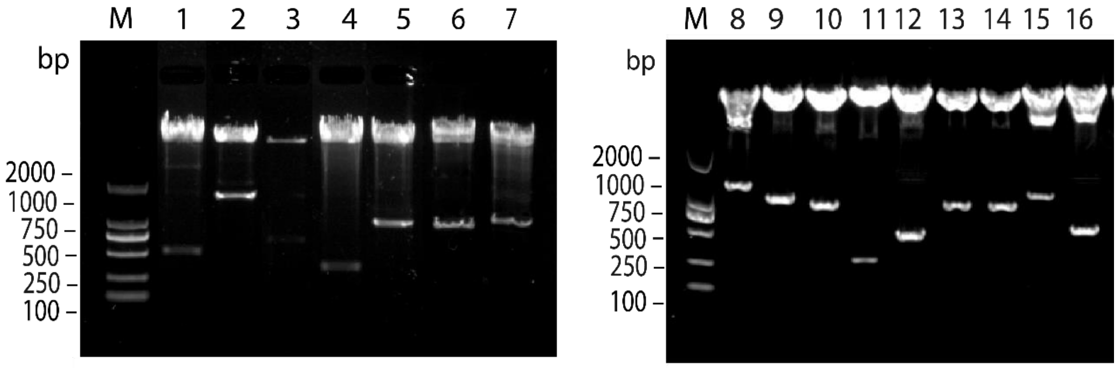

Supplementary Figure 2. Identification of eukaryotic expression plasmid by restriction enzyme analysis.

M: DNA marker; Lanes 1-16: AT730_04380; AT730_06145; AT730_07035; AT730_08185; AT730_14920; AT730_14930; AT730_14935; AT730_19120; AT730_19300; AT730_20810; AT730_20950; AT730_21050; AT730_21605; AT730_22220; AT730_22910; AT730_24225

Supplementary Table 1. Primers used for qRT-PCR analysis.

Gene	Primer	sequence(5'-3')	Product size(bp)
EF-1α	Forward	CTGGAGGCCAGCTCAAACAT	87
	Reverse	GTAATGCTAGCGGTACTACTCTTCTTGAT	
il-1b	Forward	TGGACTTCGCAGCACAAAATG	139
	Reverse	GTTCACTTCACGCTCTTGGATG	
il-4	Forward	TACATTGGTCCCCGTTTCTG	193
	Reverse	ACCCTTCAAAGCCATTCCTG	
il-8	Forward	CACGCTGTCGCTGCATTG	127
	Reverse	GTCATCAAGGTGGCAATGATCTC	
il-10	Forward	CTCTGCTCACGCTTCTTC	189
	Reverse	TCATCGTTGGACTCATAAAAC	
il-21	Forward	CTAAAGTGCTGCACCTGTCAG	181
	Reverse	TTGCACTGAGCTTTCTGTGTC	
tnf-α	Forward	ATAAGACCCAGGGCAATCAAC	177
	Reverse	CAGAGTTGTATCCACCTGTTAAATG	
inf-γ	Forward	TTCGGGATGGATGACAT	141
	Reverse	TGTAGCTGAGAATCTTCTTTAATATAG	
c3b	Forward	TGTGACCCGCTGTATGTTCT	112
	Reverse	TTGGCTGGGAAGTTCTTCAC	
tlr-1	Forward	CACCTGCGAGGAAAGTAAGT	108
	Reverse	CTGTAAGGGCGCAATCAGAC	
nf-κb	Forward	GCTCATTCAGATTGCTCTACAC	126
	Reverse	CGTGTCTCCGTTCTCATCT	
lysozyme	Forward	GACACTGGGACGCTGTGATG	174
	Reverse	AGGCCGTGCACACATAGTTG	
cox-2	Forward	TTCTTCGCCCAGCATTTCTC	181
	Reverse	AATGTGCCCCAGATCCACT	

Supplementary Table 2. Prediction of extracellular secretory proteins in *Vibrio alginolyticus* ATCC33787 by PSORTb 3.0.2

Protein name	Location score					Final predction	Secondary localization(s)
	Cytoplasmic membrane	Periplasmic	Cytoplasmic	Outer membrane	Extracellular		
AT730_00730	0.01	0.2	0	0.07	9.72	Extracellular	
AT730_01435	0.01	0.2	0	0.07	9.72	Extracellular	
AT730_02000	0	0	0	0	10	Extracellular	
AT730_03640	0	0	0	0	10	Extracellular	
AT730_03670	0.01	0.2	0.01	0.07	9.71	Extracellular	
AT730_03900	0.01	0.2	0.01	0.07	9.71	Extracellular	Host Associated
AT730_03915	0	0.04	0	1.52	8.44	Extracellular	Host Associated
AT730_03940	0.01	0.2	0.01	0.07	9.71	Extracellular	T3SS
AT730_03970	0.02	0.19	0.34	0	9.45	Extracellular	
AT730_04070	0.01	0.2	0.01	0.07	9.71	Extracellular	
AT730_04380	0.01	0.2	0.01	0.07	9.71	Extracellular	
AT730_05335	0	0	0	0	10	Extracellular	Host Associated
AT730_06145	0.01	0.11	0.01	0.23	9.64	Extracellular	
AT730_06405	0.01	0.2	0.01	0.07	9.71	Extracellular	
AT730_06925	0	0	0	0	10	Extracellular	Flagellar
AT730_06935	0	0	0	0	10	Extracellular	Flagellar
AT730_06940	0	0	0	0	10	Extracellular	Flagellar
AT730_06945	0	0	0	0	10	Extracellular	Flagellar
AT730_07035	0.01	0.11	0.01	0.23	9.64	Extracellular	
AT730_08185	0	0	0	0	10	Extracellular	Fimbrial
AT730_08220	0	0	0	0	10	Extracellular	Cytoplasmic
AT730_08640	0	0	0	0	10	Extracellular	

AT730_12975	0.02	0.19	0	0.34	9.45	Extracellular	
AT730_14210	0	0	0	0	10	Extracellular	Fimbrial
AT730_14875	0.01	0.2	0.01	0.07	9.71	Extracellular	
AT730_14880	0.01	0.2	0.01	0.07	9.71	Extracellular	
AT730_14890	0	0	0	0	10	Extracellular	Flagellar
AT730_14920	0	0	0	0	10	Extracellular	Flagellar
AT730_14930	0	0	0	0	10	Extracellular	Flagellar
AT730_14935	0	0	0	0	10	Extracellular	Flagellar
AT730_15295	0.01	0.11	0	0.23	9.65	Extracellular	
AT730_15310	0.02	0.19	0.34	0	9.45	Extracellular	
AT730_15755	0	0	0	0	10	Extracellular	
AT730_15775	0.02	0.19	0.34	0	9.45	Extracellular	
AT730_16395	0.01	0.11	0	0.23	9.65	Extracellular	
AT730_16880	0.01	0.2	0	0.07	9.72	Extracellular	
AT730_16895	0.02	0.19	0.34	0	9.45	Extracellular	
AT730_17850	0.01	0.2	0	0.07	9.72	Extracellular	
AT730_18555	0	0	0	0	10	Extracellular	
AT730_18665	0.01	0.11	0	0.23	9.65	Extracellular	
AT730_19120	0.02	0.11	0.34	0.01	9.52	Extracellular	
AT730_19205	0.01	0.2	0.01	0.07	9.71	Extracellular	
AT730_19250	0.01	0.2	0	0.07	9.72	Extracellular	
AT730_19300	0.01	0.11	0.01	0.23	9.64	Extracellular	
AT730_20490	0	0.04	0	1.52	8.44	Extracellular	Flagellar
AT730_20495	0	0	0	0	10	Extracellular	Flagellar
AT730_20515	0	0	0	0	10	Extracellular	Flagellar
AT730_20525	0	0	0	0.04	9.96	Extracellular	
AT730_20530	0.01	0.2	0.01	0.07	9.71	Extracellular	Fimbrial

AT730_20810	0.01	0.11	0.01	0.23	9.64	Extracellular	
AT730_20950	0.01	0.2	0.01	0.07	9.71	Extracellular	
AT730_21050	0.01	0.2	0.01	0.07	9.71	Extracellular	
AT730_21605	0.01	0.2	0	0.07	9.72	Extracellular	
AT730_22220	0.01	0.11	0	0.23	9.65	Extracellular	
AT730_22665	0	0	0	0	10	Extracellular	
AT730_22910	0	0	0	0	10	Extracellular	Flagellar
AT730_22920	0	0	0	0	10	Extracellular	Flagellar
AT730_22930	0	0	0	0	10	Extracellular	Flagellar
AT730_23110	0.01	0.11	0.01	0.23	9.64	Extracellular	
AT730_23410	0	0	0	0	10	Extracellular	
AT730_24225	0.01	0.11	0.01	0.23	9.64	Extracellular	
AT730_24675	0.01	0.11	0.01	0.23	9.64	Extracellular	
AT730_24680	0.01	0.11	0.01	0.23	9.64	Extracellular	
AT730_24700	0.01	0.11	0	0.23	9.65	Extracellular	
AT730_25175	0.01	0.2	0	0.07	9.72	Extracellular	
AT730_25690	0.01	0.11	0.01	0.23	9.64	Extracellular	
AT730_25735	0.01	0.2	0.01	0.07	9.71	Extracellular	
AT730_25950	0.01	0.11	0.01	0.23	9.64	Extracellular	
AT730_26235	0.01	0.11	0.01	0.23	9.64	Extracellular	

Supplementary Table 3. Information of extracellular secretory proteins in *Vibrio alginolyticus* ATCC33787

NCBI no.	Product name	Locus name	Gene name	Pred. MW（kDa）
gi|1033057234	type II toxin-antitoxin system RnlA family toxin	AT730_04380	*rnlA*	19.1
gi|1033057429	hypothetical protein	AT730_06145		63.7
gi|491522781	NAD(P)H:quinone oxidoreductase	AT730_07035	*wrbA*	20
gi|1033057543	prepilin-type N-terminal cleavage/methylation domain-containing protein	AT730_08185	*ppdD*	15.5
gi|1033057830	flagellin	AT730_14920	*fla*	40.9
gi|516430917	flagellin	AT730_14930	*fla*	40
gi|516430916	flagellin	AT730_14935	*fla*	40.5
gi|1033058117	FHA domain-containing protein	AT730_19120	*tagH*	55.1
gi|1033058135	linear amide C-N hydrolase	AT730_19300		48
gi|1033058243	baseplate J/gp47 family protein	AT730_20810		42.1
gi|960942174	thermolabile hemolysin	AT730_20950		12.3
gi|922668757	SDR family oxidoreductase	AT730_21050		26.1
gi|1033058312	lytic polysaccharide monooxygenase	AT730_21605	*lpmO*	43.5
gi|1033058344	carotenoid 1,2-hydratase	AT730_22220	*crtC*	41.8
gi|1033058400	flagellar filament capping protein	AT730_22910	*fliD*	48
gi|1033058558	hypothetical protein	AT730_24225		26.7
